# Improved Prediction of Cardiovascular Events Using Serial Cardio‐Ankle Vascular Index (CAVI) Measurements: A 10‐Year Prospective Cohort Study

**DOI:** 10.1002/clc.70382

**Published:** 2026-06-15

**Authors:** Thosaphol Limpijankit, Prin Vathesatogkit, Dujrudee Matchariyakul, Nisakorn Thongmung, Sukanya Siriyotha, Ammarin Thakkinstian, Piyamitr Sritara

**Affiliations:** ^1^ Division of Cardiology, Department of Medicine, Faculty of Medicine, Ramathibodi Hospital Mahidol University Bangkok Thailand; ^2^ Medical and Health Office, Electricity Generating Authority of Thailand, Bangkruay Nonthaburi Thailand; ^3^ Research Center, Faculty of Medicine, Ramathibodi Hospital Mahidol University Bangkok Thailand; ^4^ Department of Clinical Epidemiology and Biostatistics, Faculty of Medicine, Ramathibodi Hospital Mahidol University Bangkok Thailand

**Keywords:** arterial stiffness, cardio‐ankle vascular index, cardiovascular risk prediction, serial measurement

## Abstract

**Background:**

Most studies evaluating the cardio‐ankle vascular index (CAVI) as a marker of arterial stiffness are cross‐sectional, limiting insights into long‐term vascular changes. We investigated whether serial CAVI measurements improve the prediction of cardiovascular (CV) events beyond a single baseline value.

**Methods:**

The Electricity Generating Authority of Thailand (EGAT) study is a prospective cohort with 5‐year follow‐up intervals. Participants with prior coronary artery disease (CAD) or stroke were excluded. Demographic, clinical, laboratory data, medication use, and CAVI were collected. Serial CAVI was analyzed as a time‐varying covariate in Cox proportional hazards models, incorporating baseline, 5‐year, and 10‐year measurements. The primary composite CV outcomes comprised CAD, stroke, or CV death. Cox proportional hazards models assessed associations between baseline or serial CAVI and CV outcomes, adjusted for conventional risk factors.

**Results:**

Among 3913 participants (mean age 49.0 ± 10.8 years; 73.4% male; BMI 24.3 ± 3.6 kg/m^2^), mean CAVI increased from 7.7 ± 1.1 to 8.2 ± 1.3 over 10 years (*p* < 0.001). During a median follow‐up of 9.1 ± 3.1 years, 0.8% experienced composite CV events. Serial CAVI was independently associated with CV outcomes (HR 1.47; 95% CI 1.06–2.03; *p* = 0.019), whereas baseline CAVI was not (HR 1.14; 95% CI 0.93–1.40; *p* = 0.22). Model indices (ΔAIC = 37.1; ΔBIC = 32.7) supported superior predictive performance for serial CAVI.

**Conclusion:**

Serial CAVI measurements better predict long‐term CV events than a single baseline value. Longitudinal CAVI monitoring may enhance CV risk stratification and support preventive cardiovascular care.

## Graphical abstract

Serial CAVI measurements better predict long‐term CV events than a single baseline value, particularly for composite events, non‐fatal stroke, and CV death. In contrast, baseline CAVI showed weaker associations, with statistical significance observed only for non‐fatal CAD.

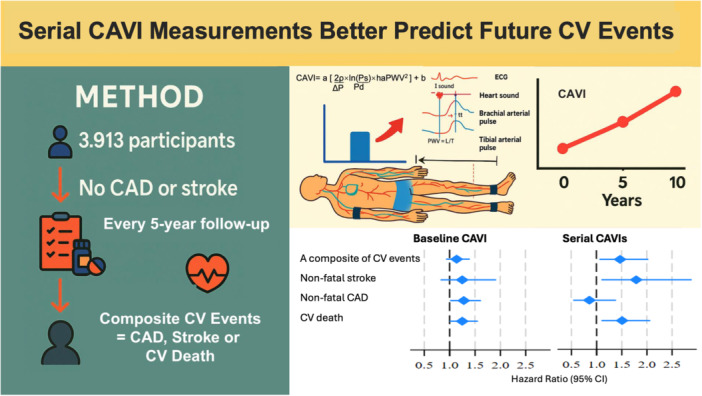

## 1 Introduction

Arterial stiffness is an important marker of cardiovascular (CV) health and has been extensively investigated for its role in subclinical atherosclerosis and in predicting future CV events [1, 2, 3]. It reflects a diminished ability of the arterial wall to accommodate pulsatile blood flow, a phenomenon that progresses with age and is accelerated by hypertension, hyperglycemia, dyslipidemia, obesity, smoking, sedentary lifestyle, and systemic inflammation [4, 5, 6, 7]. Accumulating evidence suggests that arterial stiffness is modifiable through lifestyle changes and medical therapy, with the potential to prevent target organ damage and reduce CV event rates [1, 8]. Accordingly, arterial stiffness has emerged as a valuable surrogate endpoint in both clinical practice and research.

Pulse wave velocity (PWV), particularly carotid‐femoral PWV (cfPWV), is considered the reference standard for measuring arterial stiffness [9, 10, 11]. Multiple studies have demonstratexsd that cfPWV is an independent predictor of CV morbidity and mortality in both general and high‐risk populations [12, 13, 14]. However, its interpretation is confounded by its sensitivity to blood pressure (BP) at the time of measurement, necessitating careful a'djustment [15].

The cardio‐ankle vascular index (CAVI), developed as an alternative measure of arterial stiffness, addresses some of these limitations [16, 17]. CAVI is non‐invasive, reproducible, and, unlike PWV, designed to be independent of acute BP fluctuations. Prior studies have shown that CAVI correlates strongly with subclinical atherosclerosis and is associated with incident CV events and mortality [18, 19]. Although initial data were derived primarily from Asian populations, subsequent validation in diverse cohorts has supported its broader clinical utility [20, 21].

Despite growing interest in the CAVI, most studies have relied on single‐time‐point measurements. However, arterial stiffness is dynamic and may change over time in response to therapeutic interventions or disease progression, suggesting that serial CAVI assessments could provide a more comprehensive evaluation of vascular health. To date, few studies have evaluated whether repeated CAVI measurements improve CV risk prediction compared with a single baseline assessment or how these measurements can be effectively integrated into clinical practice. As a result, important gaps remain in understanding the utility of serial CAVI monitoring for long‐term risk stratification [21, 22]. Therefore, this study aimed to determine whether serial CAVI measurements provide superior predictive value for future CV events compared with a one‐time baseline measurement.

## 2 Methods

### 2.1 Study Design and Participants

The Electricity Generating Authority of Thailand (EGAT) study is a large prospective cohort, investigating CV risk factors and disease progression among Thai adults [23]. The present analysis includes data from EGAT cohorts 1/3, 2/4, and 3/1, which were enrolled between 2007 and 2008 and followed at 5‐year intervals. The study protocol complies with the ethical principles of the Declaration of Helsinki (1975) and was approved by the Ethics Committee of the Faculty of Medicine, Ramathibodi Hospital, Mahidol University (COA.MURA2022/616). All participants provided written informed consent.

Participants were eligible for inclusion if they were free of clinically diagnosed CV disease at baseline but could have risk factors for atherosclerosis. Individuals were excluded if they had a history of coronary artery disease (CAD), stroke, or any condition that could interfere with the accuracy of CAVI measurements. Exclusion criteria included: (1) an ankle‐brachial index (ABI) < 0.9 [24], (2) interference with phonocardiogram or pulse wave acquisition, (3) atrial fibrillation, (4) outlier CAVI values (≤ 3 or ≥ 18) [25], or (5) fewer than two CAVI measurements across the follow‐up period.

### 2.2 Data Collection

Baseline data included demographic characteristics (age, sex), conventional CV risk factors (e.g., smoking status, diabetes mellitus [DM], hypertension, dyslipidemia), anthropometric measures (body mass index [BMI], waist and hip circumferences), BP, and comorbidities such as chronic kidney disease (CKD), CAD, stroke, and peripheral arterial disease (PAD).

Fasting blood samples were collected to assess plasma glucose, lipid profile (total cholesterol, high‐density lipoprotein cholesterol [HDL‐C], low‐density lipoprotein cholesterol [LDL‐C], triglycerides [TG]), uric acid, and serum creatinine. DM was defined as a fasting plasma glucose (FPG) ≥ 126 mg/dL or the use of glucose‐lowering medications. Hypertension was defined as systolic BP ≥ 140 mmHg and/or diastolic BP ≥ 90 mmHg or the use of antihypertensive therapy. Dyslipidemia was defined as total cholesterol ≥ 200 mg/dL or LDL‐C ≥ 130 mg/dL, or the use of statins. CKD was defined as estimated glomerular filtration rate (eGFR) < 60 mL/min/1.73 m^2^. Medication use was recorded, including aspirin, statins, angiotensin‐converting enzyme inhibitors (ACEIs), angiotensin receptor blockers (ARBs), beta‐blockers (BBs), calcium channel blockers (CCBs), diuretics, nitrates, and insulin. CAVI and ABI were measured at baseline and at each 5‐year follow‐up interval.

### 2.3 CAVI Measurement

**FIGURE 1:**
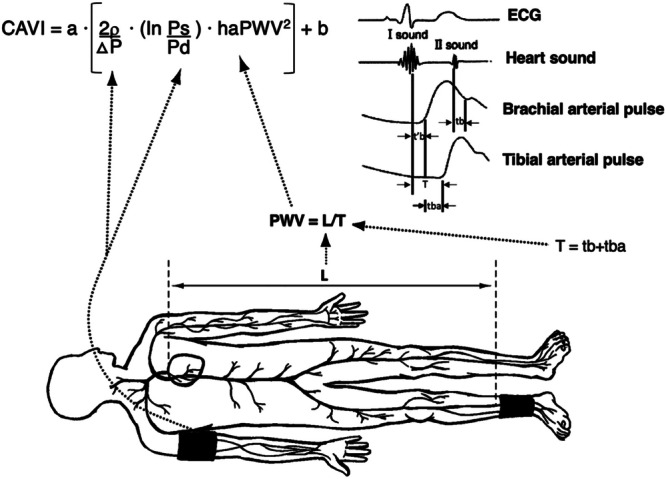
Schematic illustration of Cardio‐Ankle Vascular Index (CAVI) measurement. DP, pulse pressure (Ps – Pd); ECG, electrocardiogram; haPWV, heart–ankle pulse wave velocity; Ps, systolic blood pressure; Pd, diastolic blood pressure; ρ, blood density; tba, time between the rise of brachial and ankle pulse waves; tb, time from aortic valve closure to the dicrotic notch in the brachial pulse wave; t′b, time from aortic valve opening to the rise of the brachial pulse wave; *a* and *b*, scale conversion constants. *Source:* Modified from Shirai et al. (26).

CAVI was assessed using the Vasera VS‐1000 vascular screening system (Fukuda Denshi, Japan), which also measures ABI for PAD screening [26]. Participants rested in a supine position for 10 min before the test (Figure 1). Pressure cuffs were applied to both upper arms and ankles, and electrocardiogram (ECG) and phonocardiogram recordings were taken to assess heart rhythm and sounds. CAVI was calculated using the following formula:

CAVI = a × [{2ρ/ΔP} × ln(Ps/Pd) × haPWV^2^] + b

where Ps and Pd represent systolic and diastolic BP, respectively; ΔP = Ps − Pd; haPWV is the PWV from the heart to the ankle; ρ is blood density; and *a* and *b* are device‐specific constants. This calculation adjusts for BP to reflect arterial stiffness independent of hemodynamic fluctuations. The average of the right and left CAVI values was used for analysis. According to the manufacturer, CAVI values < 8 are considered normal, ≥ 8 to < 9 borderline, and ≥ 9 indicative of arterial stiffness [25, 27]. CAVI was measured serially at baseline and at 5‐ and 10‐year follow‐up surveys. For longitudinal data analysis, repeated measurements were incorporated as time‐varying covariates, enabling a dynamic representation of arterial stiffness exposure over time.

### 2.4 Follow‐Up and Outcomes

Following initial assessments in 2007–2008, participants were followed up at 5‐year intervals for repeat CAVI and clinical reassessment, with outcome ascertainment continuing until December 2021. Follow‐up was conducted via telephone interviews and mailed invitations. The primary outcome was a composite of major CV events, defined as non‐fatal CAD, non‐fatal stroke, or CV‐related death. The secondary outcome was all‐cause mortality. CAD was defined as any of the following: angina, acute coronary syndrome, myocardial infarction (MI), significant coronary stenosis (> 70%) on angiography, coronary revascularization (percutaneous coronary intervention or coronary artery bypass grafting), or objective evidence of ischemia on exercise testing. Stroke included ischemic or hemorrhagic stroke or transient ischemic attack. CV death was defined as death attributable to CAD or stroke. For participants with multiple events, only the first CV event was included in the analysis; individuals who died of a CV cause were classified as having both a CV event and an all‐cause death.

Vital status for non‐responders was confirmed via national databases maintained by the National Health Security Office (hospital discharge records) and the Ministry of the Interior's Department of Provincial Administration (death certificates). An independent adjudication committee comprising cardiologists and neurologists reviewed medical records and classified causes of death as CAD, stroke (including subarachnoid hemorrhage), other vascular causes (e.g., heart failure, valvular disease, PAD), non‐CV causes (e.g., malignancy, infection, accidents, or metabolic conditions), or unknown.

### 2.5 Statistical Analysis

Baseline and serial CAVI were analyzed separately to predict composite CV outcomes. Continuous variables were summarized as mean ± standard deviation (SD) or median with interquartile range (IQR), as appropriate, and categorical variables as frequencies and percentages. Group comparisons were performed using one‐way analysis of variance (ANOVA) or the Kruskal–Wallis test for continuous variables, and Chi‐square or Fisher's exact tests for categorical variables.

For baseline CAVI, Cox proportional hazard models were used to estimate its effect, with results reported as hazard ratios (HRs) and 95% confidence intervals (CIs). For serial CAVI, a time‐varying Cox model was applied, incorporating all available CAVI measurements (baseline, 5‐year, and 10‐year). This approach allowed updating each participant's risk over time, accounting for both the magnitude and timing of CAVI measurements while minimizing bias from unequal numbers of observations.

Covariate selection followed a structured two‐step approach. First, univariate Cox models were fitted for each covariate individually. Second, covariates meeting either statistical or clinical criteria were considered for inclusion in the multivariable model, which already included CAVI. Specifically, variables with *p* < 0.1 in univariate analysis or those deemed clinically important (defined as HR > 1.25 or < 0.80) were entered into the model. Forward selection based on the likelihood ratio test was then applied, and covariables with *p* < 0.05 or clinical importance were retained in the final model. Model performance was evaluated for calibration and discrimination. Calibration was assessed using a Cox–Snell residual plot, while discrimination was evaluated using Harrell's C statistic. Model performance between baseline and serial CAVI models was compared using the Akaike Information Criterion (AIC) and the Bayesian Information Criterion (BIC). The proportional hazards assumption was assessed using the global chi‐square test.

For serial CAVI, linear mixed‐effect regression models were used to identify factors associated with longitudinal CAVI measurements, accounting for within‐subject correlation from repeated measures. Covariate selection followed the same procedure as described for the Cox models, including univariate and multivariable analyses.

All statistical analyses were conducted using STATA (StataCorp, College Station, TX), with p‐values < 0.05 considered statistically significant.

## 3 Results

### 3.1 Baseline Characteristics

Between 2007 and 2008, a total of 3913 EGAT employees were enrolled in the study. Of these, 3635 participants completed the 5‐year follow‐up, and 3477 completed the 10‐year follow‐up. The cohort was predominantly male (73.4%), with a mean age of 49.0 ± 10.8 years and a mean BMI of 24.3 ± 3.6 kg/m^2^, within the normal‐to‐borderline overweight range by WHO Asian criteria (Table 1).

**TABLE 1:** Baseline and follow‐up characteristics of the study population.

Characteristics	Baseline (*n* = 3913)	5‐year F/U (*n* = 3635)	10‐year f/U (*n* = 3477)	p‐value
CAVI, mean ± SD	7.7 ± 1.1	7.8 ± 1.2	8.2 ± 1.3	< 0.001
Age, years, mean ± SD	49.0 ± 10.8	53.5 ± 10.9	59.6 ± 11.0	< 0.001
Age group, n (%)				
30 − 39	746 (19.1)	359 (9.3)	124 (3.6)	< 0.001
40 − 49	1,438 (36.7)	997 (25.8)	536 (15.5)	
50 − 59	918 (23.5)	1,350 (35.0)	1,192 (34.6)	
60 − 69	702 (17.9)	809 (21.0)	791 (22.9)	
70 − 79	109 (2.8)	346 (9.0)	807 (23.4)	
Male gender, n (%)	2,873 (73.4)	2,873 (73.4)	2,873 (73.4)	NA
BMI, kg/m^2^, mean ± SD	24.3 ± 3.6	24.7 ± 3.7	24.9 ± 3.8	< 0.001
Waist, cm, mean ± SD	87.3 ± 9.9	88.4 ± 9.9	90.1 ± 10.5	< 0.001
Hip, cm, mean ± SD	96.9 ± 6.6	97.7 ± 6.8	98.1 ± 7.5	< 0.001
SBP, mmHg, mean ± SD	124.0 ± 16.9	129.9 ± 16.7	137.8 ± 18.3	< 0.001
DBP, mmHg, mean ± SD	80.5 ± 10.6	79.3 ± 10.4	78.2 ± 10.6	< 0.001
HT, n (%)	903 (23.1)	1,377 (35.2)	1,702 (43.5)	< 0.001
DM, n (%)	293 (7.5)	516 (13.2)	699 (17.9)	< 0.001
DLP, n (%)	1,476 (37.7)	2,061 (52.7)	2,371 (64.6)	< 0.001
CKD, n (%)	157 (4.0)	376 (9.6)	607 (15.5)	< 0.001
CAD, n (%)	0 (0.0)	2 (0.1)	17 (0.4)	0.001
Stroke, n (%)	0 (0.0)	1 (0.03)	11 (0.3)	< 0.001
PAD, n (%)	5 (0.1)	8 (0.2)	33 (0.9)	< 0.001
FPG, mg/dL, mean ± SD	96.9 ± 22.8	95.4 ± 23.0	97.9 ± 25.7	< 0.001
Chol, mg/dL, mean ± SD	219.5 ± 40.1	213.5 ± 41.8	204.4 ± 42.7	< 0.001
LDL‐C, mg/dL, mean ± SD	147.0 ± 37.3	143.1 ± 38.8	135.5 ± 40.2	< 0.001
HDL‐C, mg/dL, mean ± SD	53.0 ± 13.2	58.1 ± 15.7	56.6 ± 15.3	< 0.001
TG, mg/dL, median (range)	114.0 (78.0, 163.0)	114.0 (82.0, 158.0)	113.0 (83.0, 156.0)	0.895
Uric, mg/dL, mean ± SD	5.7 ± 1.5	6.0 ± 1.5	5.9 ± 1.4	< 0.001
Creatinine, mg/dL, mean ± SD	0.9 ± 0.2	1.0 ± 0.3	1.0 ± 0.4	< 0.001
eGFR, ml/min/1.73 m^2^, mean ± SD	89.3 ± 16.9	83.9 ± 15.8	80.1 ± 17.5	< 0.001
ACEIs/ARBs, n (%)	174 (4.5)	429 (11.0)	400 (10.2)	< 0.001
Aspirin, n (%)	163 (4.2)	281 (7.2)	243 (6.2)	< 0.001
BBs, n (%)	178 (4.5)	282 (7.2)	220 (5.6)	< 0.001
CCBs, n (%)	157 (4.0)	385 (9.8)	399 (10.2)	< 0.001
Statins, n (%)	312 (8.0)	266 (6.8)	83 (2.1)	< 0.001
Diuretics, n (%)	135 (3.5)	166 (4.2)	68 (1.7)	< 0.001
Nitrates, n (%)	20 (0.5)	10 (0.3)	2 (0.1)	0.004
Insulin, n (%)	10 (0.3)	10 (0.3)	9 (0.2)	0.943

Abbreviations: ACEIs, angiotensin‐converting enzyme inhibitors; ARBs, angiotensin receptor blockers; BBs, beta‐blockers; BMI, body mass index; CAD, coronary artery disease; CAVI, cardio‐ankle vascular index; CCBs, calcium channel blockers; DBP, diastolic blood pressure; DLP, dyslipidemia; DM, diabetes mellitus; eGFR, estimate glomerular filtration rate (eGFR< 60 mL/min/1.73m^2^); FPG, fasting plasma glucose; HDL‐C, high‐density lipoprotein cholesterol; HT, hypertension; LDL‐C, low‐density lipoprotein cholesterol; PAD, peripheral arterial disease; SBP, systolic blood pressure; TG, triglyceride.

At baseline, the prevalence of dyslipidemia, hypertension, DM, and CKD was 37.7%, 23.1%, 7.5%, and 4.0%, respectively. Over time, the prevalence of these risk factors increased. The mean CAVI also increased progressively, from 7.7 ± 1.1 at baseline to 7.8 ± 1.2 at 5 years, and 8.2 ± 1.3 at 10 years (*p* < 0.001).

Regarding lifestyle changes, most participants experienced weight gain and increased waist circumference, resulting in higher BMI. Systolic BP increased, while diastolic BP decreased over the follow‐up period. FPG levels increased modestly, while lipid profiles improved overall: total cholesterol and LDL‐C decreased, HDL‐C increased, and TG levels remained stable. Renal function declined slightly, as evidenced by higher creatinine and uric acid levels and lower eGFR.

Medication use also changed significantly over time. Prescriptions for aspirin, ACEIs/ARBs, CCBs, and BBs increased. In contrast, the use of statins, diuretics, and nitrates declined, while insulin use remained unchanged.

### 3.2 Clinical Outcomes

Over a mean follow‐up period of 9.1 ± 3.1 years, 32 participants (0.8%) experienced a composite CV event, and 61 participants (1.6%) died. Among the composite events, CV deaths accounted for 0.3% (*n* = 12), non‐fatal CAD for 0.4% (*n* = 17), and non‐fatal stroke for 0.3% (*n* = 11). Non‐CV causes of death included malignancy (0.6%, *n* = 23), respiratory or gastrointestinal disease (0.1%, *n* = 4), accidents (0.1%, *n* = 5), sepsis (0.2%, *n* = 6), and other or unknown causes (0.3%, *n* = 12).

### 3.3 Predictors of CV Events

Multivariate models adjusted for baseline demographics, comorbidities, laboratory values, and medication use identified serial CAVI measurements as a significant independent predictor of composite CV events (HR: 1.47; 95% CI: 1.06–2.03; *p* = 0.019) (Table 2). There were also non‐significant trends suggesting associations between composite CV events and baseline DM (HR: 2.28; 95% CI: 0.92–5.60; *p* = 0.074) and CKD (HR: 2.53; 95% CI: 0.97–6.60; *p* = 0.058).

**TABLE 2:** Association of serial CAVI measurements with composite CV events*: univariate and multivariable Cox models.

Factors	Composite CV Events	Time at risk	Rate/1000/	Univariate		Multivariate	
	*n* = 32	(Years)	Years	HR (95% CI)	*p*‐value	HR (95% CI)	*p*‐value
Serial CAVIs	9.0 ± 1.4	—	—	1.74 (1.31, 2.31)	< 0.001	1.47 (1.06, 2.03)	0.019
HT							
Yes	20	15903.72	1.26	2.80 (1.34, 5.82)	0.006	1.59 (0.59, 4.25)	0.358
No	12	32289.69	0.37	1		1	
DM							
Yes	13	5859.11	2.22	4.15 (2.03, 8.53)	< 0.001	2.28 (0.92, 5.60)	0.074
No	19	42334.31	0.45	1		1	
CKD							
Yes	12	4555.16	2.63	4.60 (2.15, 9.88)	< 0.001	2.53 (0.97, 6.60)	0.058
No	20	43638.26	0.46	1		1	
Age	60.0 ± 8.7	—	—	1.04 (1.00, 1.08)	0.031		
Sex						
Male	29	35345.74	0.82	3.65 (1.11, 12.00)	0.033		
Female	3	12847.68	0.23	1			
BMI	24.8 ± 4.4	—	—	1.00 (0.91, 1.11)	0.941		
Waist	92.1 ± 9.9	—	—	1.03 (1.00, 1.06)	0.084		
Hip	96.3 ± 7.3	—	—	0.97 (0.92, 1.03)	0.336		
DLP							
Yes	22	23654.78	0.93	2.05 (0.93, 4.51)	0.076		
No	9	24141.93	0.37	1			
Allergy							
Yes	4	10170.29	0.39	0.92 (0.31, 2.71)	0.882		
No	28	38023.13	0.74	1			
FPG	116.8 ± 47.2	—	—	1.012 (1.006, 1.018)	< 0.001		
Total cholesterol	206.5 ± 49.0	—	—	1.00 (0.99, 1.01)	0.585		
LDL‐C	133.7 ± 44.5	—	—	1.00 (0.99, 1.01)	0.392		
HDL‐C	54.0 ± 13.9	—	—	0.98 (0.96, 1.01)	0.193		
Triglyceride	155.0 ± 95.1	—	—	1.002 (0.999, 1.005)	0.125		
Uric acid	6.2 ± 1.5	—	—	1.13 (0.88, 1.46)	0.329		
BBs							
Yes	3	2812.25	1.07	1.52 (0.46, 5.00)	0.492		
No	29	45381.17	0.64	1			
CCBs							
Yes	6	3626.91	1.65	2.35 (0.96, 5.74)	0.060		
No	26	44566.51	0.58	1			
Aspirin							
Yes	10	2810.17	3.56	6.26 (2.96, 13.25)	< 0.001		
No	22	45383.25	0.48	1			
Oral hypoglycemic drugs							
Yes	7	2828.23	2.48	3.94 (1.70, 9.15)	0.001		
No	25	45365.19	0.55	1			
Statins							
Yes	5	3818.09	1.31	1.50 (0.46, 4.94)	0.503		
No	27	44375.33	0.61	1			
Diuretics							
Yes	4	1661.13	2.41	3.77 (1.31, 10.81)	0.014		
No	28	46532.29	0.60	1			

*Note:* Abbreviations as in Table 1. *Composite CV outcomes (CV death, non‐fatal CAD, non‐fatal stroke); **AIC = 328.24; BIC = 357.34.

When stratified by specific outcomes, serial CAVI measurements were significantly associated with an increased risk of non‐fatal stroke (HR 1.79; 95% CI: 1.10–2.89; *p* = 0.018; Supplementary Table 1), but not with non‐fatal CAD (HR 0.86; 95% CI: 0.54–1.39; *p* = 0.556; Supplementary Table 2). In addition, serial CAVI was independently associated with CV death (HR 1.51; 95% CI: 1.10–2.07; *p* = 0.011; Supporting Information S1: Table 3). Other independent predictors of CV death included DM (HR 2.50; 95% CI: 1.03–6.07; *p* = 0.043) and CKD (HR 2.74; 95% CI: 1.06–7.09; *p* = 0.038).

In contrast, baseline CAVI was not significantly associated with composite CV outcomes (HR 1.14; 95% CI: 0.93–1.40; *p* = 0.222; Table 3 and Supporting Information S1: Table 4), whereas serial CAVI remained significantly associated with these outcomes (HR 1.47; 95% CI: 1.06–2.03; *p* = 0.019). DM remained a strong predictor of composite CV events in the baseline model (HR 5.86; 95% CI: 2.34–14.67; *p* < 0.001), although its effect was attenuated in the serial CAVI model (HR 2.28; 95% CI: 0.92–5.60). The effect estimates for CKD and HT were higher in the serial CAVI model than in the baseline model, although neither reached statistical significance (Table 3).

**TABLE 3:** Comparing multivariable Cox models of composite CV events between serial CAVI and baseline CAVI.

Factors	Serial CAVI		Baseline CAVI	
	HR (95% CI)	*p*‐value	HR (95% CI)	*p*‐value
CAVI	1.47 (1.06, 2.03)	0.019	1.14 (0.93, 1.40)	0.222
HT	1.59 (0.59, 4.25)	0.358	1.29 (0.53, 3.13)	0.575
DM	2.28 (0.92, 5.60)	0.074	5.86 (2.34, 14.67)	< 0.001
CKD	2.53 (0.97, 6.60)	0.058	2.06 (0.57, 7.48)	0.274

*Note:* Abbreviations as in Supporting Information S1: Table 1. Composite CV events (CV death, non‐fatal CAD, non‐fatal stroke).

Baseline CAVI was significantly associated with non‐fatal CAD (HR 1.28; 95% CI: 1.01–1.62; *p* = 0.038; Supporting Information S1: Table 5) and showed a borderline association with CV death (HR 1.25; 95% CI: 1.00–1.56; *p* = 0.055; Supporting Information S1: Table 6). No significant association was observed between baseline CAVI and non‐fatal stroke (HR 1.25; 95% CI: 0.82–1.92; *p* = 0.294; Supporting Information S1: Table 7).

### 3.4 Comparative Predictive Value of Serial Versus Baseline CAVI Measurements for CV Outcomes

**FIGURE 2:**
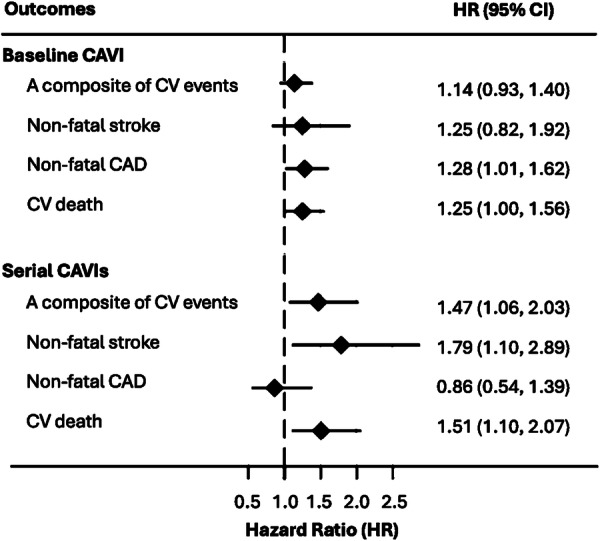
Baseline versus serial CAVI and risk of cardiovascular events: multivariable Cox proportional hazards models.

A summary of the associations between baseline and serial CAVI values and CV outcomes is presented in Table 4. Serial CAVI measurements demonstrated stronger and more consistent predictive value for most CV outcomes, particularly for composite events, non‐fatal stroke, and CV death (Figure 2). In contrast, baseline CAVI showed weaker associations, with statistical significance observed only for non‐fatal CAD.

**TABLE 4:** Comparative associations of baseline versus serial CAVI measurements with CV outcomes: multivariable Cox proportional hazards models.

Factors	Baseline CAVI		Serial CAVIs	
	HR (95% CI)	*p*‐value	HR (95% CI)	*p*‐value
Composite CV events	1.14 (0.93, 1.40)	0.222	1.47 (1.06, 2.03)	0.019
Non‐fatal stroke	1.25 (0.82, 1.92)	0.294	1.79 (1.10, 2.89)	0.018
Non‐fatal CAD	1.28 (1.01, 1.62)	0.038	0.86 (0.54, 1.39)	0.556
CV death	1.25 (1.00, 1.56)	0.055	1.51 (1.10, 2.07)	0.011

*Note:* Abbreviations as in Table 1.

Calibration goodness‐of‐fit was assessed by plotting Cox–Snell residuals against residual values. The plot demonstrated an acceptable fit at lower residual values, with some deviation observed at higher values, suggesting potential lack of fit or instability, likely due to sparse data (Supporting Information S1: Figure 1). This model showed fair discrimination between individuals with and without composite CV events, with a Harrell's C‐statistic of 0.692. The proportional hazards assumption was satisfied (global chi‐square = 1.70, df = 4, *p*‐value = 0.7916). The model incorporating serial CAVI measurements demonstrated a better fit for predicting composite CV events, with lower AIC (328.24 vs. 365.30) and BIC (357.34 vs. 390.03) values compared with the baseline CAVI model. The differences in model fit indices (ΔAIC = 37.1; ΔBIC = 32.7) further support the superior predictive performance of the serial CAVI model.

### 3.5 Exploratory Analysis of Factors Associated With CAVI Progression

An exploratory analysis was conducted to identify baseline factors associated with greater CAVI progression over time. Older age, male sex, hypertension, DM, and CKD were significantly associated with increased progression, whereas higher BMI and statin use were associated with reduced CAVI progression (Supporting Information S1: Table 8).

## 4 Discussion

In this large prospective cohort study, we demonstrated that longitudinal tracking of CAVI provides superior predictive value for long‐term CV outcomes compared with a single baseline measurement. Most previous studies have been cross‐sectional or short‐term with limited follow‐up [28, 29, 30]. Our findings address this gap, by showing that serial CAVI measurements more accurately predict future CV events, particularly non‐fatal stroke and CV death. Importantly, serial CAVI remained an independent predictor after adjustment for conventional risk factors and current treatments, whereas baseline CAVI showed only a modest association with non‐fatal stroke. These results highlight the clinical potential of incorporating serial CAVI monitoring into CV risk assessment to improve risk stratification and enable earlier, targeted interventions [31, 32].

A major strength of this study is the application of a time‐varying modeling framework, in which each repeated CAVI measurement contributes dynamically to risk estimation rather than being reduced to a single summary metric (e.g., annual average or cumulative exposure). This approach more accurately reflects longitudinal arterial stiffness burden and minimizes bias arising from unequal measurement frequency or timing across participants. Consequently, serial CAVI captures dynamic vascular changes rather than providing a static estimate.

Arterial stiffness is inherently dynamic, influenced by BP, circadian variations, diet, emotional stress, and other transient factors. A single measurement may therefore fail to capture the progression of vascular remodeling or early endothelial dysfunction. In contrast, serial CAVI assessments offer a more comprehensive evaluation of vascular health, facilitating earlier detection of subclinical atherosclerosis and supporting individualized preventive strategies [33, 34]. Longitudinal monitoring may also help assess treatment response and disease progression, aligning with principles of precision medicine.

Although alternative metrics, such as annualized changes in CAVI, may offer additional insight into the rate of arterial stiffness progression, our analysis focused on cumulative longitudinal exposure, which may better reflect the overall vascular burden over time. These approaches are likely complementary, and future studies directly comparing cumulative versus rate‐based measures are warranted to clarify their relative clinical utility, particularly in high‐risk populations who are more susceptible to rapid vascular deterioration, such as older age, male sex, hypertension, DM, and CKD.

CAVI has shown a dose‐dependent association with the coronary artery calcium score, further supporting its role in identifying individuals at elevated CV risk [35, 36]. Compared with imaging‐based modalities, CAVI offers several practical advantages: it is inexpensive, reproducible, non‐invasive, and free of radiation exposure. These features make it well‐suited for repeated use in outpatient settings [8]. In addition, visualization of longitudinal CAVI trends may enhance patient engagement and adherence to lifestyle or pharmacologic interventions [37]. Prior studies linking improvements in arterial stiffness with better clinical outcomes further support the potential clinical utility of serial monitoring [22, 38].

Important questions remain regarding the optimal implementation of serial CAVI assessment. The clinical significance of specific changes and appropriate intervention thresholds are not yet well defined. Consistent with the TRIPLE‐A‐Stiffness study [21], we analyzed CAVI as a continuous variable to preserve sensitivity to subtle vascular changes. The median annual progression in our cohort was 0.05 units, slightly lower than the 0.07 units reported previously. Although the mean increase over 10 years was modest ( + 0.5 units), each 1‐unit increase in CAVI was associated with a 47% higher risk of composite CV events and a 51% higher risk of CV death, highlighting the clinical relevance of even small long‐term increases.

A CAVI threshold > 9 has been proposed to indicate elevated CV risk [27]. However, reliance on a single measurement may overestimate long‐term risk, as it does not account for the persistence of arterial stiffness. Sustained elevations are more likely to reflect chronic vascular dysfunction and cumulative risk. Furthermore, categorical thresholds may obscure meaningful temporal trends, particularly in early disease stages when intervention may be most effective. Our findings, therefore, support the use of continuous CAVI measures for more precise risk stratification.

The overall incidence of CV events in our cohort was low (0.8% over 10 years), likely reflecting the relatively healthy baseline profile and high adherence to statin therapy and follow‐up. While this limited statistical power for some subgroup analyses, it also highlights the need for studies in higher‐risk populations or with longer follow‐up to better define the prognostic value and cost‐effectiveness of serial CAVI monitoring. At present, CAVI may be most useful for longitudinal assessment in individuals with multiple risk factors, diabetes, CKD, or suboptimal risk control, rather than as a population‐wide screening tool.

We were unable to calculate established 10‐year CVD risk scores (e.g., Framingham [39], SCORE2 [40], or ASCVD [41]) due to missing baseline variables, precluding direct comparison with standard prediction models. In addition, the relatively small number of events (*n* = 32) relative to the number of predictors raises the possibility of model overfitting (events per variable ≈ 8). Although we applied structured variable selection and evaluated model performance using calibration and discrimination metrics, some degree of overfitting cannot be excluded.

Despite these limitations, our findings suggest that CAVI captures aspects of vascular aging not fully reflected by traditional risk factors and may serve as a complementary biomarker. Larger studies with more events are needed to validate these findings and to determine whether incorporating serial CAVI improves risk discrimination beyond established models.

Future research should define optimal target populations, determine when to initiate monitoring, and clarify the relative prognostic importance of absolute CAVI values versus rates of change. Establishing appropriate follow‐up intervals will also be important. Ongoing studies, including the TRIPLE‐A‐Stiffness trial in Europe [21] and a multicenter cohort study in Japan [42], are expected to provide further insights.

In summary, our findings add to growing evidence supporting arterial stiffness as a clinically relevant and potentially modifiable risk factor. Consistent with this, the American Heart Association has recently emphasized the integration of arterial stiffness measures into CV risk assessment and management strategies [43]. Further research is needed to standardize CAVI measurement protocols, establish clinically meaningful thresholds for change, and define its role in evidence‐based CV prevention.

### 4.1 Study Limitations

The major strengths of this study include its large cohort and long‐term follow‐up. However, several limitations should be acknowledged. First, as with all longitudinal observational studies, the possibility of residual confounding remains despite extensive multivariable adjustment. Certain factors influencing the association between CAVI progression and CV outcomes may not have been measured or fully captured. Second, data on behavioral or therapeutic adherence were not systematically collected during follow‐up, precluding assessment of the impact of lifestyle modifications or medication changes on arterial stiffness progression. Third, our cohort consisted predominantly of male EGAT employees of relatively homogeneous ethnic background, which may limit the generalizability of the findings to women, younger individuals, and more ethnically diverse populations. Fourth, the small number of events raises concern for model overfitting; although we used parsimonious modeling and sensitivity analyses, these findings should be interpreted cautiously and validated in larger cohorts. Finally, the absence of key baseline variables precluded calculation of established risk scores, limiting comparison of incremental predictive value.

## 5 Conclusion

Serial CAVI measurements, modeled as time‐varying covariates, provide greater predictive accuracy for CV events than a single baseline assessment. Incorporating repeated evaluations of arterial stiffness into clinical practice may enhance long‐term risk prediction, facilitate earlier intervention, and support monitoring of treatment response. CAVI should be considered a complementary tool rather than a replacement for established risk models. Further studies are needed to validate these findings and to define optimal strategies for integrating serial CAVI monitoring into CV risk assessment.

## Author Contributions

**Thosaphol Limpijankit:** conceptualizations, methodology, study design, data analysis, interpretation, and manuscript writing. **Prin Vathesatogkit:** data acquisition, data analysis, interpretation, and study supervision. **Dujrudee Matchariyakul:** data acquisition and data analysis. **Nisakorn Thongmung:** data acquisition. **Sukanya Siriyotha:** data acquisition, data analysis, and interpretation. **Ammarin Thakkinstian:** data analysis, interpretation, and manuscript revision. **Piyamitr Sritara:** study supervision. All authors read and approved the final manuscript.

## Conflicts of Interest

The authors declare no conflicts of interest.

## Supporting information

Supporting File

## Data Availability

The data that support the findings of this study are not openly available due to reasons of sensitivity and are available from the corresponding author upon reasonable request. Data are stored in a controlled‐access data repository at the Department of Clinical Epidemiology and Biostatistics, Faculty of Medicine, Ramathibodi Hospital, Mahidol University, Bangkok, Thailand.
