# Supplementary material for: Improved Prediction of Cardiovascular Events Using Serial Cardio‐Ankle Vascular Index (CAVI) Measurements: A 10‐Year Prospective Cohort Study
Source: Clin Cardiol. 2026 Jun 15;49(6):e70382. doi: 10.1002/clc.70382 (PMC13267990; doi:10.1002/clc.70382)

**Supplementary Table 1.** **Association of Serial CAVI Measurements with Non-Fatal Stroke: Univariate and Multivariable Cox Models.**

| Factors | Univariate | | Multivariate | |
| --- | --- | --- | --- | --- |
|  | HR (95% CI) | P-value | HR (95% CI) | P-value |
| Serial CAVIs | 1.91 (1.28, 2.85) | 0.001 | 1.79 (1.10, 2.89) | 0.018 |
| FPG | 1.02 (1.01, 1.03) | <0.001 | 1.01 (1.00, 1.02) | 0.001 |
| Age | 1.00 (0.95, 1.06) | 0.972 |  |  |
| BMI | 1.00 (0.85, 1.18) | 0.960 |  |  |
| Waist | 1.03 (0.97, 1.09) | 0.331 |  |  |
| Hip | 0.97 (0.9, 1.05) | 0.513 |  |  |
| HT | 2.44 (0.69, 8.58) | 0.164 |  |  |
| DM | 2.86 (0.82, 9.96) | 0.099 |  |  |
| DLP | 1.86 (0.48, 7.19) | 0.370 |  |  |
| CKD | 3.56 (0.97, 13.08) | 0.055 |  |  |
| Allergy | 2.66 (0.68, 10.46) | 0.160 |  |  |
| Total cholesterol | 1.00 (0.98, 1.01) | 0.578 |  |  |
| LDL-C | 0.99 (0.97, 1.01) | 0.229 |  |  |
| HDL-C | 1.00 (0.96, 1.04) | 0.969 |  |  |
| Triglyceride | 1.002 (0.999, 1.005) | 0.069 |  |  |
| Uric acid | 1.10 (0.71, 1.69) | 0.680 |  |  |
| Calcium channel blockers | 2.03 (0.43, 9.45) | 0.368 |  |  |
| Aspirin | 3.05 (0.66, 14.17) | 0.155 |  |  |
| Oral hypoglycemic drugs | 4.64 (1.22, 17.61) | 0.024 |  |  |
| Statins | 2.47 (0.64, 9.62) | 0.191 |  |  |

BMI: body mass index, CAVIs: cardio-ankle vascular indices, CCBs: Calcium channel blockers, CKD: chronic kidney disease, DLP: dyslipidemia, DM: diabetes mellitus, FPG: fasting plasma glucose, HT: hypertension, LDL-C: low-density lipoprotein cholesterol, HDL-C: high-density lipoprotein cholesterol.

**Supplementary Table 2.** Association of Serial CAVI Measurements with Non-Fatal CAD: Univariate and Multivariable Cox Models.

| Factors | Univariate | | Multivariate | |
| --- | --- | --- | --- | --- |
|  | HR (95% CI) | P-value | HR (95% CI) | P-value |
| Serial CAVIs | 0.94 (0.57, 1.53) | 0.791 | 0.86 (0.54, 1.39) | 0.556 |
| Diuretics | 12.17 (3.9, 38.02) | <0.001 | 14.02 (3.20, 61.48) | <0.001 |
| Aspirin | 7.48 (2.76, 20.26) | <0.001 | 6.90 (1.69, 28.14) | 0.007 |
| Age | 0.99 (0.95, 1.03) | 0.619 |  |  |
| Male | 2.72 (0.62, 11.88) | 0.184 |  |  |
| BMI | 1.05 (0.92, 1.19) | 0.507 |  |  |
| Waist | 1.03 (0.98, 1.07) | 0.292 |  |  |
| Hip | 1.00 (0.93, 1.07) | 0.894 |  |  |
| HT | 1.76 (0.66, 4.67) | 0.258 |  |  |
| DM | 2.44 (0.90, 6.65) | 0.081 |  |  |
| DLP | 1.11 (0.41, 3.03) | 0.839 |  |  |
| CKD | 1.70 (0.54, 5.37) | 0.365 |  |  |
| Allergy | 1.11 (0.25, 4.88) | 0.887 |  |  |
| FPG | 1.01 (1.00, 1.02) | 0.178 |  |  |
| Total cholesterol | 1.01 (1.00, 1.02) | 0.101 |  |  |
| LDL-C | 1.01 (1.00, 1.02) | 0.127 |  |  |
| HDL-C | 0.98 (0.94, 1.02) | 0.254 |  |  |
| Triglyceride | 1.001 (0.998, 0.005) | 0.437 |  |  |
| Uric acid | 1.02 (0.71, 1.47) | 0.899 |  |  |
| Beta blockers | 3.21 (0.92, 11.19) | 0.067 |  |  |
| Calcium channel blockers | 2.65 (0.86, 8.12) | 0.089 |  |  |
| Oral hypoglycemic drugs | 3.59 (1.17, 11.03) | 0.025 |  |  |
| Statins | 1.21 (0.35, 4.24) | 0.763 |  |  |

Abbreviations as in Supplementary Table 1.

**Supplementary Table 3.** **Association of Serial CAVI Measurements with CV Death: Univariate and Multivariable Cox Models**.

| Factors | Univariate | | Multivariate | |
| --- | --- | --- | --- | --- |
|  | HR (95% CI) | P-value | HR (95% CI) | P-value |
| Serial CAVIs | 1.34 (0.81, 2.22) | 0.259 | 1.51 (1.10, 2.07) | 0.011 |
| DM | 5.44 (1.63, 18.17) | 0.006 | 2.50 (1.03, 6.07) | 0.043 |
| CKD | 2.99 (0.84, 10.60) | 0.090 | 2.74 (1.06, 7.09) | 0.038 |
| Age | 0.88 (0.76, 1.03) | 0.106 |  |  |
| Male | 2.16 (0.47, 9.89) | 0.320 |  |  |
| BMI | 0.88 (0.72, 1.07) | 0.199 |  |  |
| Waist | 0.99 (0.93, 1.06) | 0.874 |  |  |
| Hip | 0.92 (0.83, 1.01) | 0.094 |  |  |
| FPG | 1.01 (0.99, 1.03) | 0.452 |  |  |
| Total cholesterol | 0.99 (0.98, 1.01) | 0.507 |  |  |
| LDL-C | 1.00 (0.98, 1.02) | 0.855 |  |  |
| HDL-C | 0.96 (0.92, 1.01) | 0.146 |  |  |
| Triglyceride | 1.00 (0.99, 1.01) | 0.865 |  |  |
| Uric acid | 1.53 (0.96, 2.43) | 0.071 |  |  |
| Beta blockers | 4.30 (0.93, 19.91) | 0.062 |  |  |
| Calcium channel blockers | 0.97 (0.12, 7.55) | 0.976 |  |  |
| Aspirin | 4.36 (1.18, 16.15) | 0.027 |  |  |
| Oral hypoglycemic drugs | 1.23 (0.16, 9.54) | 0.846 |  |  |
| Statins | 0.58 (0.07, 4.51) | 0.601 |  |  |

Abbreviations as in Supplementary Table 1.

**Supplementary Table 4.** Association of Baseline CAVI with Composite CV Events*: Univariate and Multivariable Cox Models*.*

| Factors | Composite CV Events | Time at risk | Rate/1000 | Univariate | | Multivariate | |
| --- | --- | --- | --- | --- | --- | --- | --- |
|  | n=32 | (Years) | (Years) | HR (95% CI) | P-value | HR (95% CI) | P-value |
| Baseline CAVIs | 8.6±1.2 | − | − | 1.36 (1.18, 1.56) | <0.001 | 1.14 (0.93, 1.40) | 0.222 |
| HT |  |  |  |  |  |  |  |
| Yes | 16 | 11,558.79 | 1.38 | 2.91 (1.44, 5.89) | 0.003 | 1.29 (0.53, 3.13) | 0.575 |
| No | 16 | 36,634.63 | 0.44 | 1 |  | 1 |  |
| DM |  |  |  |  |  |  |  |
| Yes | 12 | 3,733.85 | 3.21 | 6.60 (3.20, 13.63) | <0.001 | 5.86 (2.34, 14.67) | <0.001 |
| No | 20 | 44,459.57 | 0.45 | 1 |  | 1 |  |
| CKD |  |  |  |  |  |  |  |
| Yes | 5 | 2,044.25 | 2.45 | 3.70 (1.40, 9.76) | 0.008 | 2.06 (0.57, 7.48) | 0.274 |
| No | 27 | 46,149.16 | 0.59 | 1 |  | 1 |  |
| Age | 54.9±7.5 | − | − | 1.04 (1.01, 1.08) | 0.025 |  |  |
| Sex |  |  |  |  |  |  |  |
| Male | 29 | 35,345.74 | 0.82 | 3.65 (1.11, 12.00) | 0.033 |  |  |
| Female | 3 | 12,847.68 | 0.23 | 1 |  |  |  |
| BMI | 25.3±4.1 | − | − | 1.07 (0.98, 1.17) | 0.152 |  |  |
| Waist | 92.0±10.4 | − | − | 1.04 (1.01, 1.08) | 0.011 |  |  |
| Hip | 96.7±6.5 | − | − | 1.00 (0.94, 1.05) | 0.899 |  |  |
| DLP |  |  |  |  |  |  |  |
| Yes | 21 | 18,566.41 | 1.13 | 2.85 (1.37, 5.95) | 0.005 |  |  |
| No | 11 | 29,627.01 | 0.37 | 1 |  |  |  |
| Allergy |  |  |  |  |  |  |  |
| Yes | 6 | 16,056.84 | 0.37 | 0.51 (0.21, 1.25) | 0.140 |  |  |
| No | 26 | 32,136.58 | 0.81 | 1 |  |  |  |
| FPG | 112.1±38.2 | − | − | 1.012 (1.005, 1.020) | 0.001 |  |  |
| Total cholesterol | 222.6±41.6 | − | − | 1.002 (0.993, 1.010) | 0.612 |  |  |
| LDL-C | 147.7±36.5 | − | − | 1.001 (0.991, 1.010) | 0.853 |  |  |
| HDL-C | 53.4±12.9 | − | − | 0.999 (0.973, 1.030) | 0.955 |  |  |
| Triglyceride | 147.6±102.8 | − | − | 1.001 (0.998, 1.000) | 0.387 |  |  |
| Uric acid | 5.9±1.8 | − | − | 1.09 (0.83, 1.42) | 0.540 |  |  |
| BBs |  |  |  |  |  |  |  |
| Yes | 1 | 2,374.01 | 0.42 | 0.53 (0.07, 3.93) | 0.535 |  |  |
| No | 31 | 45,819.4 | 0.68 | 1 |  |  |  |
| CCBs |  |  |  |  |  |  |  |
| Yes | 1 | 2,081.79 | 0.48 | 0.57 (0.08, 4.27) | 0.588 |  |  |
| No | 31 | 46,111.63 | 0.67 | 1 |  |  |  |
| Aspirin |  |  |  |  |  |  |  |
| Yes | 3 | 2,147.33 | 1.40 | 1.93 (0.58, 6.43) | 0.283 |  |  |
| No | 29 | 46,046.08 | 0.63 | 1 |  |  |  |
| Oral hypoglycemic drugs |  |  |  |  |  |  |  |
| Yes | 3 | 1,818.14 | 1.65 | 2.23 (0.67, 7.44) | 0.193 |  |  |
| No | 29 | 46,375.28 | 0.63 | 1 |  |  |  |
| Statins |  |  |  |  |  |  |  |
| Yes | 1 | 537.81 | 1.86 | 2.92 (0.4, 21.39) | 0.292 |  |  |
| No | 31 | 47,655.6 | 0.65 | 1 |  |  |  |
| Diuretics |  |  |  |  |  |  |  |
| Yes | 3 | 1,789.89 | 1.68 | 2.24 (0.67, 7.50) | 0.190 |  |  |
| No | 29 | 46,403.53 | 0.63 | 1 |  |  |  |
|  |  |  |  |  |  |  |  |

Abbreviations as in Supplementary Table 1.

*Composite CV events (CV death, non-fatal CAD, non-fatal stroke)

**Compared to the serial CAVIs model, AIC and BIC were higher (365.30 vs. 328.24 and 390.03 vs. 357.34, respectively.

**Supplementary Table 5.** Association of Baseline CAVI with Non-Fatal CAD: Univariate and Multivariable Cox Models.

| Factors | Univariate | | Multivariate | |
| --- | --- | --- | --- | --- |
|  | HR (95% CI) | P-value | HR (95% CI) | P-value |
| Baseline CAVI | 1.34 (1.10, 1.63) | 0.004 | 1.28 (1.01, 1.62) | 0.038 |
| Diuretics | 5.39 (1.54, 18.86) | 0.008 | 2.81 (0.56, 13.88) | 0.205 |
| Aspirin | 2.75 (0.63, 12.09) | 0.180 | 0.83 (0.10, 6.85) | 0.860 |
| DM | 6.41 (2.37, 17.37) | <0.001 |  |  |
| CKD | 4.66 (1.33, 16.3) | 0.016 |  |  |
| Age | 1.03 (0.98, 1.08) | 0.190 |  |  |
| Male | 2.72 (0.62, 11.88) | 0.184 |  |  |
| BMI | 1.07 (0.95, 1.21) | 0.268 |  |  |
| Waist | 1.04 (1.00, 1.09) | 0.066 |  |  |
| Hip | 1.02 (0.95, 1.09) | 0.585 |  |  |
| HT | 2.18 (0.83, 5.75) | 0.114 |  |  |
| DLP | 2.25 (0.85, 5.92) | 0.101 |  |  |
| Allergy | 0.46 (0.13, 1.63) | 0.229 |  |  |
| FPG | 1.002 (0.984, 1.02) | 0.805 |  |  |
| Total cholesterol | 1.004 (0.993, 1.02) | 0.466 |  |  |
| LDL-C | 1.003 (0.990, 1.020) | 0.651 |  |  |
| HDL-C | 1.001 (0.966, 1.04) | 0.967 |  |  |
| Triglyceride | 1.001 (0.996, 1.01) | 0.751 |  |  |
| Uric acid | 1.06 (0.75, 1.51) | 0.725 |  |  |
| Beta blockers | 1.15 (0.15, 8.72) | 0.891 |  |  |
| Calcium blockers | 1.34 (0.18, 10.12) | 0.779 |  |  |
| Oral hypoglycemic drugs | 3.28 (0.75, 14.42) | 0.116 |  |  |

Abbreviations as in Supplementary Table 1.

**Supplementary Table 6.** Association of Baseline CAVI with CV Death: Univariate and Multivariable Cox Models.

| Factors | Univariate | | Multivariate | |
| --- | --- | --- | --- | --- |
|  | HR (95% CI) | P-value | HR (95% CI) | P-value |
| Baseline CAVI | 1.38 (1.13, 1.69) | 0.002 | 1.25 (1.00, 1.56) | 0.055 |
| DM | 8.24 (2.61, 26.03) | <0.001 | 6.46 (1.92, 21.72) | 0.003 |
| FPG | 1.02 (1.01, 1.03) | <0.001 |  |  |
| HT | 4.30 (1.36, 13.58) | 0.013 |  |  |
| Waist | 1.06 (1.01, 1.11) | 0.025 |  |  |
| Age | 1.07 (1.01, 1.13) | 0.026 |  |  |
| CKD | 4.20 (0.91, 19.25) | 0.065 |  |  |
| DLP | 3.09 (0.93, 10.29) | 0.066 |  |  |
| Male | 1.84 (0.40, 8.38) | 0.433 |  |  |
| BMI | 1.11 (0.96, 1.28) | 0.170 |  |  |
| Hip | 1.01 (0.92, 1.10) | 0.888 |  |  |
| LDL-C | 0.99 (0.98, 1.01) | 0.490 |  |  |
| HDL-C | 1.00 (0.95, 1.04) | 0.916 |  |  |
| Triglyceride | 1.002 (0.998, 1.010) | 0.279 |  |  |
| Uric acid | 0.90 (0.54, 1.50) | 0.694 |  |  |
| Aspirin | 1.80 (0.23, 14.03) | 0.573 |  |  |
| Oral hypoglycemic drugs | 2.16 (0.28, 16.79) | 0.463 |  |  |

Abbreviations as in Supplementary Table 1.

**Supplementary Table 7.** Association of Baseline CAVI with Non-Fatal Stroke: Univariate and Multivariable Cox Models.

| Factors | Univariate | | Multivariate | |
| --- | --- | --- | --- | --- |
|  | HR (95% CI) | P-value | HR (95% CI) | P-value |
| Baseline CAVI | 1.39 (1.14, 1.71) | 0.001 | 1.25 (0.82, 1.92) | 0.294 |
| FPG | 1.02 (1.01, 1.03) | <0.001 | 1.02 (1.01, 1.03) | 0.003 |
| Statins | 9.10 (1.16, 71.11) | 0.035 |  |  |
| DM | 6.57 (1.91, 22.52) | 0.003 |  |  |
| HT | 3.67 (1.11, 12.09) | 0.033 |  |  |
| DLP | 4.14 (1.10, 15.63) | 0.036 |  |  |
| Age | 1.03 (0.97, 1.09) | 0.301 |  |  |
| BMI | 1.04 (0.88, 1.22) | 0.676 |  |  |
| Waist | 1.04 (0.98, 1.10) | 0.251 |  |  |
| Hip | 0.96 (0.88, 1.06) | 0.449 |  |  |
| CKD | 2.08 (0.26, 16.34) | 0.487 |  |  |
| Allergy | 0.50 (0.11, 2.38) | 0.386 |  |  |
| Total cholesterol | 0.997 (0.982, 1.010) | 0.708 |  |  |
| LDL-C | 0.996 (0.979, 1.010) | 0.624 |  |  |
| HDL-C | 1.005 (0.960, 1.050) | 0.846 |  |  |
| Triglyceride | 1.002 (0.996, 1.010) | 0.510 |  |  |
| Uric acid | 0.95 (0.62, 1.44) | 0.802 |  |  |
| Oral hypoglycemic drugs | 2.36 (0.30, 18.56) | 0.416 |  |  |

Abbreviations as in Supplementary Table 1.

**Supplementary Table 8.** Baseline factors associated with CAVI progression: A univariate and multivariate multilevel mixed-effects linear regression analysis.

| Factors | Univariate | | | Multivariate | |
| --- | --- | --- | --- | --- | --- |
|  | mean ± SE | Coef (95% CI) | P-value | Coef (95% CI) | P-value |
| Age, 10 years | − | 0.59 (0.58, 0.61) | <0.001 | 0.56 (0.54, 0.58) | <0.001 |
| Sex |  |  |  |  |  |
| Male | 7.98 (0.02) | 0.28 (0.21, 0.35) | <0.001 | 0.29 (0.24, 0.34) | <0.001 |
| Female | 7.70 (0.03) | 0 |  | 0 |  |
| BMI | − | -0.025 (-0.033, -0.017) | <0.001 | -0.05 (-0.06, -0.04) | <0.001 |
| HT |  | 0 |  | 0 |  |
| Yes | 8.28 (0.02) | 0.58 (0.53, 0.63) | <0.001 | 0.12 (0.07, 0.16) | <0.001 |
| No | 7.71 (0.02) | 0 |  | 0 |  |
| DM |  |  |  |  |  |
| Yes | 8.46 (0.04) | 0.64 (0.57, 0.71) | <0.001 | 0.28 (0.22, 0.35) | <0.001 |
| No | 7.82 (0.02) | 0 |  | 0 |  |
| CKD |  |  |  |  |  |
| Yes | 8.58 (0.04) | 0.75 (0.68, 0.83) | <0.001 | 0.12 (0.06, 0.19) | <0.001 |
| No | 7.83 (0.02) | 0 |  | 0 |  |
| Statin |  |  |  |  |  |
| Yes | 8.10 (0.03) | 0.22 (0.15, 0.29) | <0.001 | -0.02 (-0.18, -0.06) | <0.001 |
| No | 7.88 (0.02) | 0 |  | 0 |  |
| DLP |  |  |  |  |  |
| Yes | 8.12 (0.02) | 0.45 (0.40, 0.49) | <0.001 |  |  |
| No | 7.68 (0.02) | 0 |  |  |  |
| Allergy |  |  |  |  |  |
| Yes | 7.71 (0.02) | -0.24 (-0.29, -0.20) | <0.001 |  |  |
| No | 7.95 (0.02) | 0 |  |  |  |
| Beta-blockers |  |  |  |  |  |
| Yes | 8.04 (0.05) | 0.14 (0.06, 0.23) | 0.002 |  |  |
| No | 7.89 (0.02) | 0 |  |  |  |
| Calcium channel blockers |  |  |  |  |  |
| Yes | 8.13 (0.04) | 0.25 (0.18, 0.33) | <0.001 |  |  |
| No | 7.88 (0.02) | 0 |  |  |  |
| Diuretics |  |  |  |  |  |
| Yes | 8.07 (0.06) | 0.18 (0.06, 0.30) | 0.004 |  |  |
| No | 7.90 (0.02) | 0 |  |  |  |
| Aspirin |  |  |  |  |  |
| Yes | 8.12 (0.05) | 0.03 (0.14, 0.32) | <0.001 |  |  |
| No | 7.89 (0.02) | 0 |  |  |  |
| Oral hypoglycemic drugs |  |  |  |  |  |
| Yes | 8.20 (0.05) | 0.32 (0.23, 0.41) | <0.001 |  |  |
| No | 7.88 (0.02) | 0 |  |  |  |

Abbreviations as in Supplementary Table 1.

**Supplementary Figure 1.** Assessing goodness of fit of the multivariable Cox model for serial CAVI


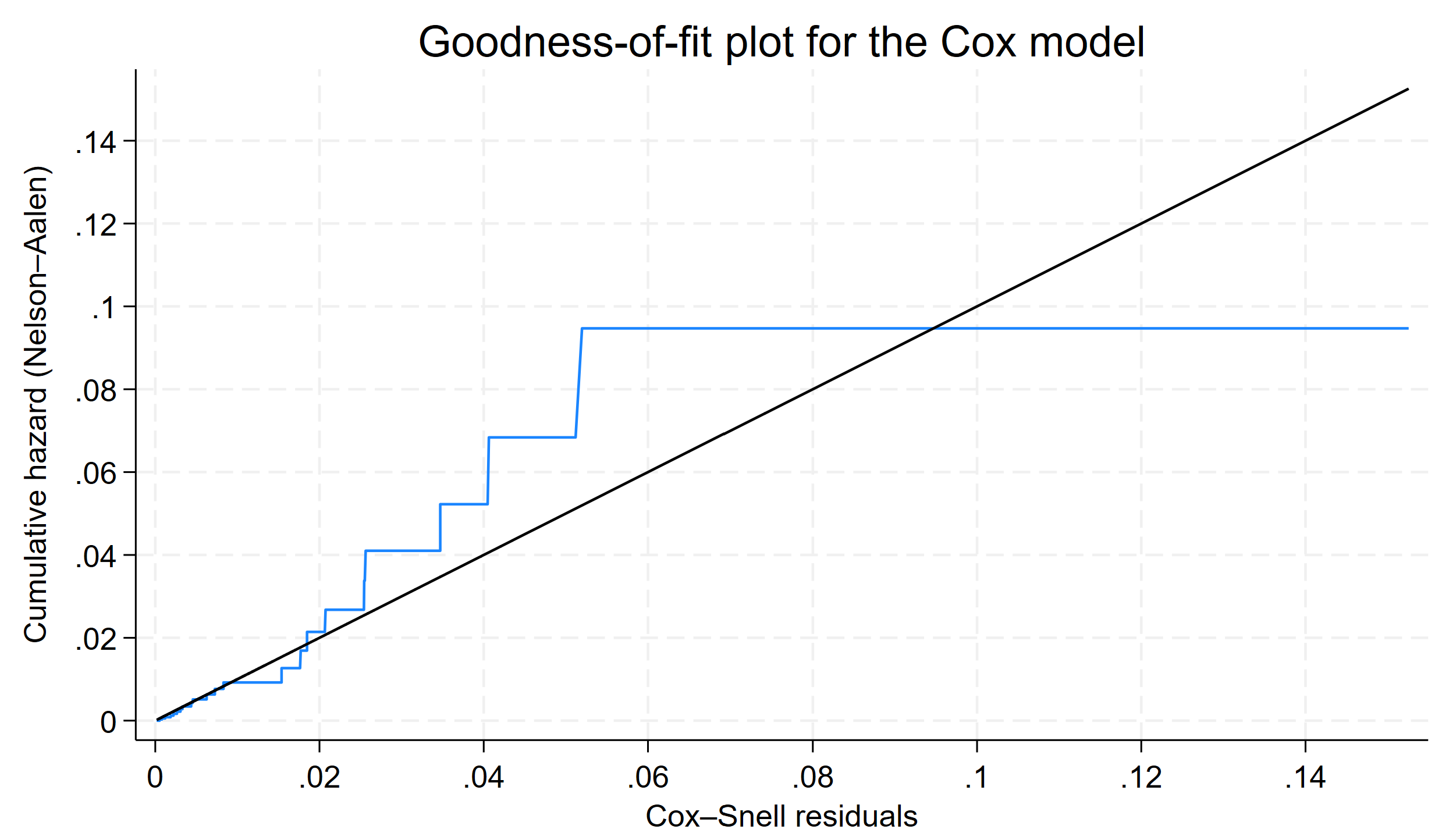

Supplement: Supplementary file 1 — Supporting File [file CLC-49-e70382-s001.docx]
